# EHMTI-0288. A clinical comparison demonstrates similarities between chronic and high frequency episodic migraine

**DOI:** 10.1186/1129-2377-15-S1-E28

**Published:** 2014-09-18

**Authors:** P Pozo-Rosich, M Quintana, M Torres, J Fernandez-Morales, J Alvarez-Sabin

**Affiliations:** 1 Neurology, Hospital Universitari Vall d'Hebron, Barcelona, Spain; 2 Headache Research Lab, Vall d'Hebron Research Institute, Barcelona, Spain

## 

To do a clinical comparison in migraine according to the number of headache days suffered per month

According to the IHCD-III beta, migraine can be classified as episodic migraine (intuitively) and chronic migraine (CM). 1109 patients were analyzed and divided into three different groups: low-frequency episodic migraine (LFEM: 0-9 days/month), high-frequency episodic migraine (HFEM: 10-14 days/months) and chronic migraine (CM≥15 days/month). Clinical characteristics (sociodemographics, comorbid diseases, frequency, treatments, disability -MIDAS-, impact -HIT6-, anxiety -STAI-, and depression -BDI- were analyzed using first an univariate analysis and then a logistic regression model.

Differences have been found between the LFEM-HFEM and LFEM-CM. Between LFEM-HFEM, three risk factors have been observed associated in an independent manner: medication overuse [OR=19.21;IC(5.50-67.05);p<0.001], MIDAS scale disability [OR=1.47;IC(1.24-1.74);p<0.001] and the presence of compressive type pain [OR=1.77;IC(1.21-2.59);p<0.05]. Between LFEM and CM, three independent risk factors have been independently associated with CM: medication overuse [OR=20.74;IC(3.07-140.23);p<0.05], BDI-II scale grade [OR=1.77;IC(1.24-2.54);p<0.05] and verbal analysis of pain. No differences were found between HFEM and CM except for the frequency and duration of the attacks.

CM is defined as suffering from headache and migraine on 15 or more days. However, this division is arbitrary. Our study demonstrates that CM and HFEM are practically the same, and that LFEM is different from HFEM and CM. This creates reasonable doubts regarding the chronification of migraine and when to consider a patient to be chronic. We propose that HFEM patients be considered as CM.

No conflict of interest.

